# The status in Africa of fall armyworm expressing genetic markers related to infestations of pasture, millet, alfalfa, and rice in the Americas

**DOI:** 10.1371/journal.pone.0329096

**Published:** 2025-07-31

**Authors:** Rodney N. Nagoshi

**Affiliations:** Center for Medical, Agricultural and Veterinary Entomology, United States Department of Agriculture-Agricultural Research Service, Gainesville, Florida, United States of America; National Cheng Kung University, TAIWAN

## Abstract

The establishment of the fall armyworm (FAW), *Spodoptera frugiperda*, in Africa is reported to have caused substantial agricultural losses since its detection in 2016 and subsequent spread into Asia and Australia by 2020. Based on the crops being infested, it appears that the population (C strain) primarily responsible for FAW infestations of corn in the Americas is widespread in Africa but there is uncertainty about the status of the R strain that targets pastures, alfalfa, millet, and rice in the United States. The two strains can only physically be distinguished by molecular markers, with single nucleotide polymorphisms (SNPs) in the mitochondrial Cytochrome oxidase subunit I (*COI*) and nuclear Z-chromosome-linked Triosephosphate isomerase (*Tpi*) gene demonstrated to consistently identify strains in both American continents. However, the *COI* and *Tpi* markers are generally in disagreement in the Eastern Hemisphere. This together with conflicting results from whole genome SNP studies creates uncertainty about the strain composition of this invasive population. In this paper the legitimacy of the *Tpi* markers is supported and used to not only confirm the existence of the R strain in Africa, but to also provide evidence for the introduction of new R strain variants in western Africa since 2017. These findings have implications on the crops at risk in the Eastern Hemisphere and for understanding how the invasion of Africa by FAW occurred.

## Introduction

In 2016, established populations of *Spodoptera frugiperda* (J. E. Smith) (Lepidoptera: Noctuidae), commonly called fall armyworm (FAW), were discovered in western Africa [1]. This important pest of the Americas was then detected in most of sub-Saharan Africa by 2018 and much of central and southeastern Asia and Australia by 2020 [2]. FAW is one of the primary pests of corn in the Americas and can cause significant yield losses in several other crops. As such, it represents a major economic threat to the Eastern Hemisphere with potential economic losses estimated to be in the billions of USD [3,4]. However, more recent observations suggest that FAW is displacing other pests and so the net increase in crop losses may be lower than expected [5–9].

The Western Hemisphere origin of the current African FAW population remains speculative, though our past work suggests that Florida or the Caribbean are the most likely candidates. This is based on polymorphisms in the mitochondrial *Cytochrome oxidase subunit I* gene (*COI*) that produce a set of haplotypes designated here as *COI*-CSh(1–4) [10]. These haplotypes show reproducible differences in relative frequencies that divide Western Hemisphere FAW into two geographically defined groups. The “FL-type” haplotype profile is found in most of the Caribbean islands, Florida, and the annual migratory destinations of the Florida population that extends northward along the U.S. eastern seaboard up to the state of Pennsylvania [11]. The rest of the hemisphere is of the “TX-type”, which include populations overwintering in Texas, Mexico and South America. Our initial survey of specimens from 2016–2018 found the FAW in Africa, India, Myanmar, and China to be consistently of the FL-type [12–14]. An exception to this pattern was found after more extensive surveys in 2018 when collections in Benin, Ghana, and Togo showed transient expression of the TX-type CSh profile [15]. This was interpreted as potentially arising from a new introduction of FAW with a probable source outside the Florida-Caribbean region.

The risk of infestation by FAW in specific crops is influenced by the distribution of two FAW populations historically designated as “host strains” that are defined by their differential distribution on host plants in the field, with the C strain the majority in corn and sorghum while the R strain predominating in alfalfa, millet, and pasture habitats [16–18]. Numerous feeding studies have failed to find consistent significant differences in the fitness of the strains on different plant species, suggesting that this phenotype is based more on a “preference” rather than nutritional necessity (reviewed in [19]). Strain determination is made difficult by their being morphologically indistinguishable, restricting identification to molecular methods [20]. A further complication is the observation of limited but consistently observed cross-hybridization between the strains both in the laboratory [21,22] and in the field [23], consistent with the suggestion that these are at an intermediate stage of speciation [18,24,25]. Significant gene flow between the strains is in agreement with other studies concluding that the FAW in North America [26] and most of the rest of the Western Hemisphere [27] behave as a panmictic population.

These observations have important implications to our conceptions of the FAW strains. Gene flow between strains will tend to limit the development of strain-specific phenotypes and if the magnitude of this genetic exchange varies by location, then the types of such phenotypes might differ. This appears to be the case as differential host use is the only strain-specific phenotype demonstrated in populations from both American continents, while the strain specificity initially reported for allochronic mating behavior [22,28–31] and female pheromones [32,33] have turned out to be regionally limited [34,35].

Similar variability was found with genetic markers mapping to autosomes, which explains inconsistencies in strain identification between several studies using whole genome SNP comparisons. The primary reason why some whole genome studies identified strains (e.g., [36–38]) while others did not (e.g., [25,27,39,40]) appears to be the proportion of Z-chromosome SNP markers, which is where the preponderance of strain-specific SNPs mapped. While a few autosomal SNPs displayed strain-specificity, there is no evidence that any were identified in more than one study. This lack of reproducibility suggests that the specificity of these autosomal SNPs is regional rather than universal. These concerns indicate that putative strain markers need to at minimum be validated by demonstrations of strain specificity in FAW populations from both Americas. I believe the evidence is most consistent with strain identity being primarily, if not solely, Z-linked [25,41], with only nuclear markers mapping to the Z-chromosome currently able to universally identify the FAW host strains (reviewed in [19,25]).

The best characterized and most used strain markers are polymorphisms in the mitochondrial *COI* and in exon segments of the *Z*-chromosome-linked *Triosephosphate isomerase* (*Tpi*) gene [42–44]. Specifically, FAW expressing the C strain-associated alleles (C_COI_ and C_Tpi_) are usually from corn and sorghum hosts while R strain polymorphisms (R_COI_ and R_Tpi_) predominate in specimens collected from pasture and forage grasses, alfalfa, millet, and sporadically in rice [10,16,17,20]. The current working hypothesis is that the strain specificity of the *Z*-linked *Tpi* markers is due to their physical linkage to the genes determining strain behavior, while the specificity of the *COI* markers results from the chance asymmetric distribution of mitochondrial haplotypes between the strains. This hypothesis can explain the surprising finding that the strain markers mostly disagree in the Eastern Hemisphere [23,45,46], where the majority of FAW outside of western Africa express the discordant R_COI_ C_Tpi_ configuration [12–14]. This was suggested to have resulted from hybridization between the two strains during their invasion of Africa that led to an “unlinking” of the mitochondrial and *Z*-chromosome strain-specific haplotypes [46]. A similar mechanism was proposed to explain observations of a significant R_COI_ C_Tpi_ population in Western Hemisphere field surveys [17,18,23,47], with its plausibility supported by the generation of the same discordant marker configuration in the laboratory through the directed cross-hybridization between strains [44]. In this scenario, *Tpi* remains a valid global strain marker while *COI* is not reliable for Eastern Hemisphere populations [12]. Recent papers have proposed similar explanations for the discrepancy between the *COI* and *Tpi* strain markers, including interstrain hybridization [38] or that R_COI_ C_Tpi_ FAW from the Western Hemisphere were part of the invasive propagule [48,49].

It should be noted however that polymorphisms in only two segments of the *Tpi* gene have been tested for their correspondence to the diagnostic host plant use phenotype in populations from both Americas. These are the above described *Tpi* exon segment, TpiE4 [42–44], and the adjacent intron fragment, TpiI4a200 [41]. The full-length *Tpi* coding sequence was recently used to discriminate the host strains, and it was suggested that the greater number of polymorphisms available should make for more accurate identification [50]. However, the correspondence of this longer sequence to host plant use was not demonstrated nor was there any attempt to assess sequence variability within the strain groupings. These deficiencies make the strain identification of haplotypes that do not group near the small number of reference sequences tested uncertain and arbitrary.

Our earlier identification that 2% of African specimens were of the R strain was based on the diagnostic gTpi183Y SNP in a *Tpi* exon that identified a single R strain variant designated AfrRa1 [51]. However, this strain identification should be considered preliminary as AfrRa1 carries additional exon polymorphisms not found in a survey of over 400 specimens from Argentina, Brazil, Florida, and Texas [45,51]. AfrRa1 was initially observed in 11 specimens from five African countries (Togo, Ghana, the Democratic Republic of the Congo, Kenya, and South Africa) and it was detected as a heterozygote with C_Tpi_ in specimens from India and Myanmar [12–14]. Analysis of a *Tpi* intron segment (TpiI4) that was shown to be highly variable in Western Hemisphere populations found only a single sequence in all AfrRa1 specimens, making it likely that AfrRa1 as defined by gTpi183Y represents a single *Tpi* gene variant [52].

The AfrRa1 *Tpi* exon sequence are identical to another line reported as “Africa-specific” or “Zambia strain” found in collections from African and China [50,53]. The Zambia strain was originally characterized from an inbred colony derived from 2017 field collections in Zambia corn and was shown by whole genome analysis to carry a combination of what was characterized as C strain-related and R strain-related SNPs, with a higher proportion of the former [53]. These observations have led to proposals that the Zambia haplotype may be evolving independently in Africa from the C strain population but is not of the R strain, thereby potentially representing a population distinct from the traditional strains [50,53]. However, given concerns about the consistency of whole genome approaches to strain identification this conclusion should be considered preliminary.

This study is part of an effort to describe the genetic composition of the African FAW population in the years immediately after its first discovery and its presumed introduction into the continent, with the twin objectives of better understanding how the invasion took place and to provide a genetic baseline from which new introductions can be detected. With respect to the latter, this an extension of earlier research from this laboratory suggesting that such an incursion occurred in 2018 in western Africa [15]. Additional collections as well as more extensive sequence analysis were performed to test previous conclusions and to examine whether this putative second introduction of FAW into western Africa in 2018 resulted in the presence of new FAW haplotypes in Africa. One consequence of the extended genetic survey is new insight on the strain identity of the AfrRa1 line and the Zambia strain by phylogenic analysis. These results are relevant to the current threat of the R strain to African agriculture and the likelihood of future introductions of new R strain variants from the Western Hemisphere that could increase that risk.

## Materials and methods

### Description of FAW collections

The focus of this study is on the African FAW composition during the years immediately after the presumed introduction circa 2016. For this reason, collections were limited to the 2016−2019 period. The FAW collections analyzed were all described in previous published studies (Table 1). The processes specimens were kept in storage as DNA at −20°C, while excess specimens from each collection were stored in alcohol under refrigeration. Specimens were re-sequenced and, in some cases, additional specimens from these collections were analyzed for *COI* or *Tpi* as needed. Collections from the Mexican states of Chiapas (2023) and Durango (2016, 2017) tested for the presence of AfrRa1 were obtained from N.M. Rosas Garcia and E.A.M. Rivera as part of a separate study. The Chiapas specimens were from larvae collected from corn then raised in the laboratory with the F1 used for the genetic analysis. The Durango larvae were field collections from corn.

**Table 1:** Description of FAW collections.

Country (collection year)	In-country Location	Collection^1^	Reference
South America: Brazil (2005), Argentina (2012), Peru (2014), Bolivia (2012), Ecuador (2018−9), Paraguay (2006)	Multiple	L	[10,17,54]
Mexico (2013–2017)	Multiple	L	[55,56]
Texas (2004–2011)	Multiple	T	[11,56,57]
Florida (2012–2016)	Multiple	T	[56,58]
Caribbean: Puerto Rico (2016), Trinidad (2013), Barbados (2015)	Multiple	T	[13,58]
Africa (year, abbreviation)			
Benin (2017–2019, Ben17–19)	Setto	L	[15]
Burundi (2016–2017, Bur16–17)	Multiple	L	[45]
Cabo Verde (2017, CV17)	Multiple	L	[12]
Central African Republic (2017, CAR17)	Multiple	L	[12]
Chad (2017, Cha17)	Multiple	L	[12]
Comoros (2018, Com18)	Mohéli	L	[15]
Dem. Rep. of Congo(south) (2017, DRCs17)	Haut-Katanga	L	[45]
Dem. Rep. of Congo(north) (2017, DRCn17)	Sud-Ubangi	L	[45]
Ethiopia (2017, Eth17)	Awash Melkasa	L	[15]
Gabon ((2018, Gab18)	Multiple	L	[15]
Ghana (2016–2018, Gha16–18)	Multiple	L	[12,15,59]
Kenya (2017, Ken17)	Multiple	L	[45]
Nigeria (2016, Nig16)	Ibadan	L	[15]
Republic of the Congo (2018, Cng18)	Apoko	L	[15]
Sao Tome (2016, SaT16)	Multiple	L	[45]
Senegal (2018–2019, Sen18–19)	Sangalkam	L	[15]
South Africa (2017−2019, SAf17-19)	Multiple	L + T	[12,13,15]
Tanzania (2017, 2019, Tan17–19)	Morogoro	L	[15,45]
Togo (2016–2019, Tog16–19)	Multiple	L	[51,59,60]
Zambia (2017, Zam17)	Serenje	L	[45]
Zimbabwe (2017, Zim17)	Harare	L	[15]
Asia (year, abbreviation)			
India (2018, Ind18)	Karnathaka	L	[13]
Myanmar (2019, Mya19)	Multiple	L	[14]
China (2019, Chi19)	Multiple	L	[14]

^1^ L=Larval collections, T=pheromone trap.

### DNA sequence analysis

Nuclear and mitochondrial DNA were isolated from single specimens by homogenization in a 5-ml Dounce homogenizer (Thermo Fisher Scientific, Waltham, Massachusetts, USA) in 1 ml of phosphate buffered saline (PBS, 20 mM sodium phosphate, 150 mM NaCl, pH 8.0). The homogenate was transferred to a 2-ml microcentrifuge tube and pelleted by centrifugation at 6000 g for 5 minutes at room temperature. The pellet was resuspended in 400 µl Genomic Lysis buffer (Zymo Research, Orange, California, USA) and incubated at 55°C in a dry bead bath for at least three hours. Debris was removed by centrifugation at 10,000 rpm for 5 minutes. The supernatant was transferred to a Zymo-Spin II or Zymo-Spin III column (Zymo Research, Orange, California, USA) and processed according to manufacturer’s instructions.

The relevant segments from the *COI* and *Tpi* genes were amplified by polymerase chain reaction (PCR) amplification in a 30-µl reaction mix containing 3 µl of 10X manufacturer’s reaction buffer, 1 µl 10mM dNTP, 0.5 µl 20-µM primer mix, 1 µl DNA template (between 0.05–0.5 µg), 0.5 units Taq DNA polymerase (New England Biolabs, Beverly, Massachusetts) with the remaining volume water. The thermocycling program was 94°C (1 min), followed by 30 cycles of 92°C (30 s), 56°C (45 s), 72°C (45 s), and a final segment of 72°C for 3 min. Primers used for the PCR amplification of COIB are c891F (5’-TACACGAGCATATTTTACATC-3’) and c1472R (5’-GCTGGTGGTAAATTTTGATATC-3’) [56]. Primers for the *Tpi* segment used for strain identification and that includes the TpiI4a200 intron fragment are t412F (5’-CCGGACTGAAGGTTATCGCTTG-3’) and t1140R (5’-GCGGAAGCATTCGCTGACAACC-3’) [41].

The PCR products were separated by agarose gel electrophoresis and purified using the Zymoclean Gel DNA Recovery Kit (Zymo Research, Orange, California). The isolated fragments were directly analyzed by DNA sequencing performed by either Azenta Life Sciences (Chelmsford, Massachusetts) or Eurofins Genomics (Louisville, Kentucky). All primers were synthesized by Integrated DNA Technologies (Coralville, Iowa).

### Characterization of the FAW strains using genetic markers

**Fig 1:**
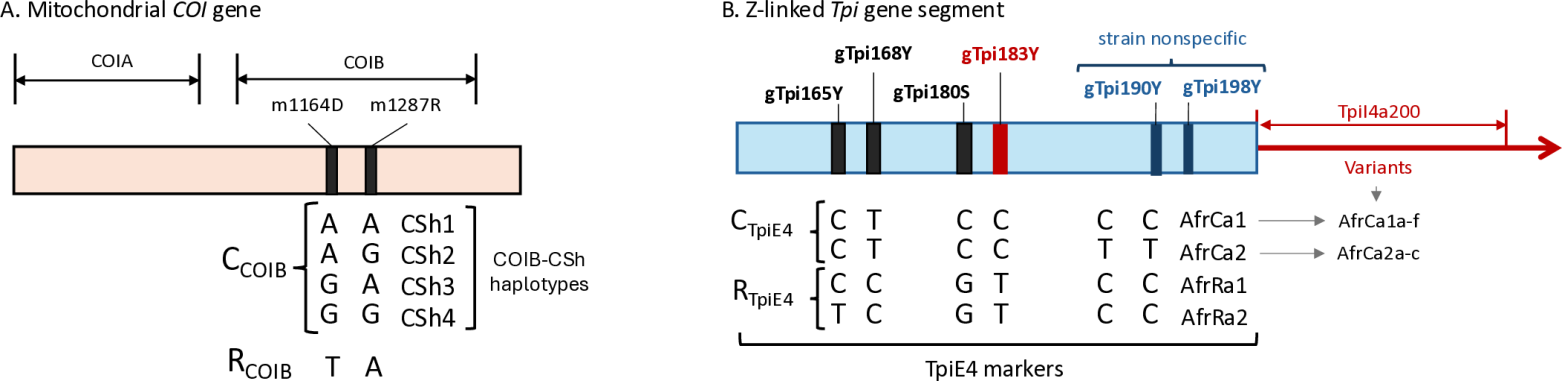
Description of the COIB and TpiE4 haplotype markers. A, molecular map of the *COI* gene. COIA is region frequently used for DNA barcoding. The locations of the m1164D and m1287R polymorphisms in COIB are indicated with the empirically observed nucleotides listed below. The T_1164_A_1287_ combination identifies the R-strain based on *COI* (R_COI_) while the other combinations define the COIB-based C-strain haplotypes (CSh) that are designated CSh1-h4. B, molecular map of the TpiE4 and TpiI4a200 segments indicating the relative locations SNPs that define the African FAW TpiE4 haplotypes. TpiI4a200 variants are denoted by a lower-case letter added to the linked TpiE4 haplotype with the DNA sequence described in S1 Fig.

COIB describes an approximately 500-bp segment of the *COI* gene that lies downstream of the region typically used for DNA barcoding (Fig 1A). The COIB-CSh haplotypes are derived from two polymorphic sites, mCOI1164D and mCOI1287R. The mCOI1164D site contains either an A, G, or T while mCOI1287R varies between A or G. The configuration T_1164_A_1287_ is diagnostic of the R strain (R_COIB_) and four variants define the C strain (C_COIB_), A_1164_A_1287_ (CSh1), A_1164_G_1287_ (CSh2), G_1164_A_1287_ (Csh3), G_1164_G_1287_ (CSh4) [61].

The fourth exon of the *Tpi* gene (TpiE4) contains three polymorphic sites (gTpi165Y, gTpi168Y, and gTpi183Y) that can serve as host strain markers in Africa FAW populations, with gTpi183Y showing the most consistent correspondence with the host strain phenotype (Fig 1B) [23,44]. The R strain is defined by a T at gTpi183Y (R_TpiE4_) while a C denotes the C strain (C_TpiE4_). The intron following this exon, TpiI4, displays high sequence variation and is of variable size because of frequent indels [41,62]. To simplify comparisons, the sequence analysis was limited to a portion of the first 60% of the intron [46]. An earlier study using a subset of the collections from 2016–2017 from 11 African countries found only seven TpiI4a200 haplotypes, six associated with the C_TpiE4_ group (AfrCa1a, AfrCa1b, AfrCa1c, AfrCa2a, AfrCa2b, and AfrCa2c) and one found in all R_TpiE4_ specimens (AfrRa1). An additional 128 sequences from these collections were added in the current study as well as 51 sequences from four additional countries (Nigeria, Benin, Cabo Verde, and Ethiopia) surveyed during the 2016–2017 period. As standard protocol, sequence variations that were observed only once were reanalyzed by one or more repeat PCR amplifications and DNA sequencing. This was done to reduce the likelihood of including artifacts resulting from amplification and sequencing errors.

### Identification of C_TpiE4_/R_TpiE4_ strain hybrids

*Tpi* is *Z*-linked and so present in two copies in males. Because the PCR product amplified from the nuclear genome is directly sequenced, heterozygosity for polymorphisms within the region of interest will produce ambiguous sequence data in the form of overlapping chromatogram curves [23]. Heterozygosity identified by this method at the strain diagnostic gTpi183Y SNP were classified as interstrain hybrids (H_TpiE4_).

### Phylogenetic analysis of TpiI4a200

Phylogenetic trees were produced with Geneious Prime 2021.1.1 software [63] using the Maximum Likelihood [64] method. All analyses underwent bootstrap testing (100 replicates) with the optimal tree shown and drawn to scale. The evolutionary distances were computed using the Maximum Composite Likelihood method [65].

### Analysis and presentation of data

DNA sequence analysis including alignments and phylogeny were performed using Geneious Prime 2021.1.1 (Biomatters, Auckland, New Zealand). The generation of graphs were done using Excel and PowerPoint (Microsoft, Redmond, WA). Sequences used in the phylogenetic analysis have been deposited into GenBank. All DNA haplotypes described are available in the NCBI Genbank database. TpiI4a200 haplotypes used in the phylogenetic analysis: Argentina sequences (Accession numbers ON586157-ON586213), Brazil sequences (ON586214 - ON586265), Florida sequences (ON586266 - ON586329). Africa TpiI4a200 haplotypes (includes portion of TpiE4 exon sequence): AfrCa1a (PV125350), AfrCa1b (PV125351), AfrCa1c (PV125352), AfrCa1d (PV125358), AfrCa1e (PV125356), AfrCa1f (PV125357), AfrCa2a (PV125353), AfrCa2b (PV125354), AfrCa2c (PV125355), AfrRa1 (PV125359), AfrRa2 (PV125360). All geographical maps were made with Natural Earth using QGIS version 3.24 (Open Source Geospatial Foundation). QGIS is created and distributed under the GNU General Public License (GPL) and the current documentation is provided under the GNU Free Documentation License, Version 1.3 or any later version.

## Results

### *Testing the* COI*-CSh haplotype shift in Africa*

**Fig 2:**
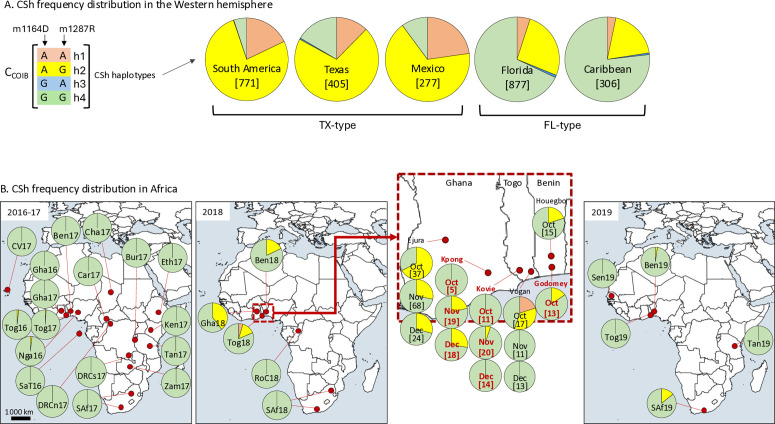
Distribution of the COIB-CSh haplotypes. A, distribution of CSh haplotypes at different Western Hemisphere locations. B, distribution of CSh haplotypes in multiple locations and times in Africa. Text in red indicate new sequence data from this study. Geographical maps with Natural Earth using QGIS version 3.24 (Open Source Geospatial Foundation).

The COIB-CSh haplotypes are defined by two polymorphic sites in the COIB segment of the *COI* gene that has been used to identify the FAW C (C_COIB_) and R strains (R_COIB_) (Fig 1A) [10,57]. The C_COIB_ population can be further divided into four COIB-CSh groups (CSh1-h4), with persistent differences in the proportions of CSh2 and CSh4 subdividing Western Hemisphere FAW into two groups (Fig 2A, [10]). The CSh1 variant is a minority haplotype that is detectable in all four regions at between 3% to 18%, while CSh3 is rarely observed, ranging in frequency from 1% to 3%.

Nagoshi et al. (2022) suggested a new introduction of FAW into western Africa based on changes in the COIB-CSh haplotype frequencies observed in 2018 [15]. Most African locations surveyed in 2016–2019 were dominated by R_COIB_, with the minority C_COIB_ population almost entirely of the CSh4 haplotype. For example, 99% of all African C_COIB_ specimens were CSh4 (323/327), with all exceptions CSh2 and from Togo (Fig 2B). However, there was an influx of CSh2 into Ejura, Ghana in Oct-Nov 2018 (CSh2/total = 51/129), both CSh1 and CSh2 in Vogan, Togo in Oct 2018 (11/23), and of CSh2 in Houegbo, Benin in Oct 2018 (3/15). This study examined three additional sites during 2018 in the region with evidence of increased CSh2 frequency in two locations, in Nov-Dec 2018 in Kpong, Ghana (9/37) and in Oct 2018 in Godomey, Benin (2/13) but less so in Kovie, Togo (inset, Fig 2B).

The near absence of CSh2 and complete absence of Csh1 observed in 2016 and 2017 across the continent followed by their transient and localized uptick in 2018 is most consistent with an introduction of FAW from outside Africa that then dissipates into the much larger endogenous population by 2019. In this scenario, Texas, Mexico or South America are possible sources as these are locations where CSh1 and CSh2 are most frequent [15].

### The TpiI4a200 segment identified new FAW variants in Africa in 2018

**Fig 3:**
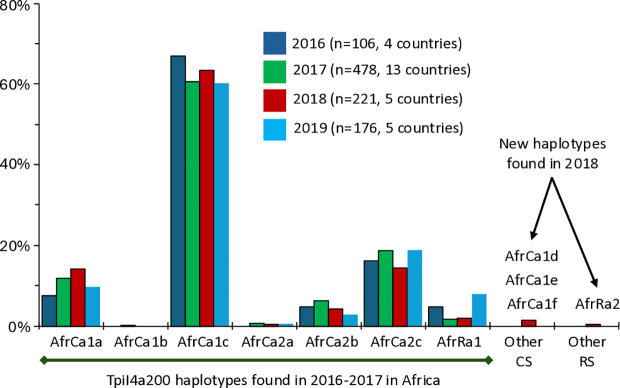
Distribution of the TpiI4a200 variants in Africa from 2016-2019. The frequencies of the TpiI4a200 haplotypes observed in collections from 2016-2017 are shown for collections from 2016-2019. In addition, new variants found in 2018 are described. The locations of the collection sites are as mapped in Fig 2B. Capture numbers can be found in S1 Table.

An expectation of a major new introduction of FAW would be the addition of new *Tpi* variants. This was tested by examining the African collections with the *Tpi* intron segment TpiI4a200, which shows high genetic variation in the Western Hemisphere. This is indicated by the 106 sequence variants found from an examination of 273 Western Hemisphere specimens [41]. In this study, the TpiI4a200 sequence from 584 specimens collected from 16 African nations in 2016 and 2017 identified only seven TpiI4a200 haplotypes (Fig 3). The same seven haplotypes were observed in 2018 (221 specimens) and 2019 (176 specimens), with no statistically significant differences found based on Pearson *R* values ≥ 0.99 for all pairwise comparisons. This occurred despite differences in the number and locations of the collection sites between years, suggesting general homogeneity for this marker across much of the African continent.

In 2018 single specimens of four new TpiI4a200 variants were identified from a sampling of 221 specimens (“Other” CS and RS, Fig 3). Three were associated with C_TpiE4_ (AfrCa1d, e, f) and one with R_TpiE4_ (AfrRa2) and all were from the October 2018, Vogan, Togo collection. The coincidence between the influx of the CSh1 and CSh2 with the detection of new *Tpi* alleles is suggestive that they arose from the same event.

### Determination of strain identity by TpiI4a200 phylogenetic analysis

**Fig 4:**
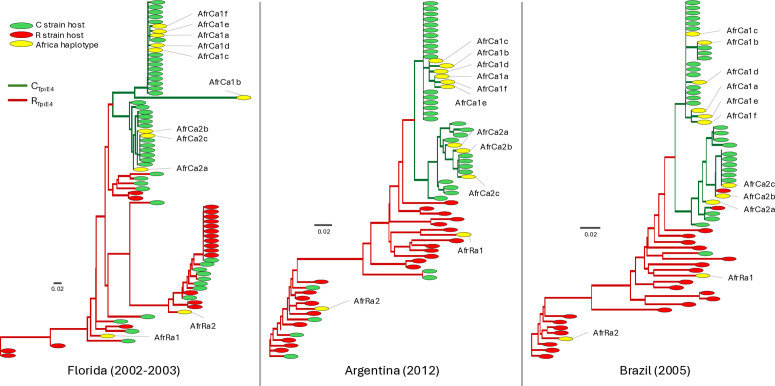
Maximum-Likelihood phylogenetic trees produced using the TpiI4a200 segment from Florida, Brazil, and Argentina collections (midpoint rooting). Clades were attributed to strains according to TpiE4, with C (green lines) and R (red lines) strain clades determined by the Tpi183Y SNP. Colored ovals identify the strain category of the host plant from which the sample was collected (red or green), while yellow ovals identify the African TpiI4a200 haplotypes. The scale bar represents 0.02 nucleotide substitutions per site.

It was previously demonstrated that a Maximum-Likelihood phylogenetic tree based on the TpiI4a200 sequence identifies phylogenetic groups that corresponds to both the *COI* and TpiE4 strain markers and to host plant usage [41]. This potentially provides a more accurate strain identification method than the single gTpi183Y SNP marker previously used. The eleven African TpiI4a200 haplotypes were separately compared to 75 sequences from Florida, 63 from Brazil, and 57 from Argentina that were derived from field collected larvae from either C strain or R strain preferred host plants (Fig 4). Clades were categorized by the gTpi183Y exon marker as C_TpiE4_ (green lines) or R_TpiE4_ (red lines) strain and the host plants associated with each specimen indicated. The correspondence between host plant and the TpiE4-based phylogenetic groupings was generally high in all three countries for the C_TpiE4_ strain. For example, only two C_TpiE4_ specimens out of 87 (both from Brazil) were found on an R strain associated host plant. Greater variability was found with R_TpiE4_ specimens, where 28% (24/85) were found on C strain associated hosts. This was mostly due to the Florida population, where the R strain may be behaving as a generalist with respect to host use [19,41].

In all three phylogenetic trees, the African haplotypes fell within the phylogenetic groups consistent with their gTpi183Y strain identification. Seven of the African C_TpiI4_ haplotypes were identical to sequences found in the Western Hemisphere. The three exceptions were all from the group identified in 2018 (AfrCa1d, e, f) and formed a cluster with AfrCa1a. Both AfrRa1 and AfrRa2 were singletons, which is not unusual for R_TpiI4_ haplotypes because of the high genetic variation in the TpiI4a200 intron (Fig 4).

### *Identifying R*_*TpiE4*_*/C*_*TpiE4*_ *hybrids*

**Fig 5:**
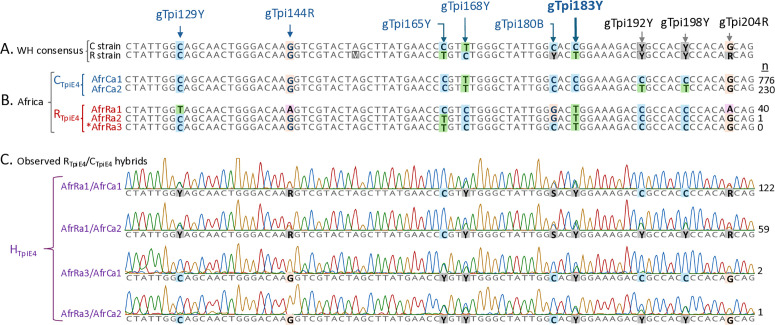
TpiE4 sequences and chromatographs showing relevant SNPs. The consensus Western Hemisphere TpiE4 sequences for two strains are WH C strain and WH R strain, with gTpi165Y, gTpi168Y, and gTpi183Y the strain specific SNPs. Polymorphic nomenclature follows IUPAC convention, M = A/C, R = A/G, Y = C/T. Number of samples analyzed (n) is given for the various African TpiE4 haplotypes and hybrids observed. The AfrRa3 sequence is extrapolated from heterozygotes.

To better understand the distribution of the infrequent R_TpiI4_ haplotypes an attempt was made to identify R_TpiI4_/C_TpiI4_ hybrids. The TpiE4 exon segment contains three SNPs that show strain-specific variation (gTpi165Y, gTpi168Y, and gTpi183Y) in Western Hemisphere populations, with gTpi183Y used as the diagnostic strain marker (Fig 1B). The Western Hemisphere consensus sequences for the two strains share two strain nonspecific SNP sites at gTpi192Y and gTpi198Y as well as additional polymorphic sites relative to the African haplotypes (Fig 5). The eleven TpiI4a200 African haplotypes fall into four TpiE4 categories, two each for the C_TpiE4_ strain and R_TpiE4_ strain. The two C_TpiE4_ groups are distinguished by gTpi192Y and gTpi198Y each carrying a C (the AfrCa1 group) or a T (AfrCa2 group). The AfrCa1 and AfrCa2 groups can be further subdivided by variations in the TpiI4a200 intron segment, but these are not shown in Fig 5.

The AfrRa1 exon differs from the Western Hemisphere consensus R strain sequence at gTpi165Y and introduces new polymorphisms at gTpi129Y, gTpi144R, and gTpi180B. The AfrRa2 allele differs from AfrRa1 at gTpi129Y, gTpi144R, and gTpi165Y, where it is like the Western Hemisphere R strain consensus sequence, but it still retains the unusual gTpi180B polymorphism (Fig 5).

Overlapping C/T chromatograph peaks at the strain-specific SNPs identify R_TpiE4_/C_TpiE4_ males produced by hybridization between the strains and were designated as H_TpiE4_ (Fig 5). four hybrid configurations were identified. The most common were the patterns associated with AfrRa1 as a heterozygote with either a AfrCa1 or AfrCa2 *Z*-chromosome. No hybrids with AfrRa2 have been found to date. The two remaining hybrid patterns suggest the presence of a third R_TpiE4_ variant (AfrRa3) that resembles the Western Hemisphere R strain consensus sequence in that the strain-specific exon sites are limited to gTpi165Y, gTpi168Y, and gTpi183Y. The three AfrRa3 hybrid specimens were from a South Africa 2019 collection.

### Distribution of the R strain in Africa and Asia

**Fig 6:**
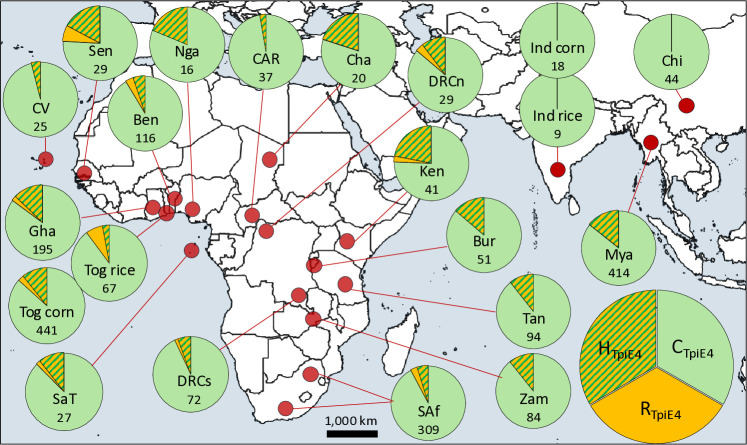
Distribution of the C_TpiE4_, R_TpiE4_ and H_TpiE4_ populations in the Eastern Hemisphere. Pie charts show proportions of each strain and hybrids for each collection. The approximate locations of the collection sites are denoted by red circles. Capture numbers are available in S2 Table. The number of specimens examined in each collection is listed in the circle. Geographical map was made with Natural Earth using QGIS version 3.24 (Open Source Geospatial Foundation).

The R strain as identified by R_TpiE4_ was found as a homozygote or hemizygote in only 3% (41) of the pooled 1,786 specimens from 11 African countries. In no individual country did this group make up more than 7% of the population [12]. In this study heterozygotes, [C/R]_TpiE4_, were included to provide a more comprehensive estimate of the frequency of R strain *Z*-chromosomes. About 12% (185/1,786) of the pooled African population carried at least one R_TpiE4_ *Z*-chromosome, with the great majority of these AfrRa1. The R_TpiE4_ *Z*-chromosome was broadly distributed across the African continent with no obvious regional differences (Fig 6). Most of the R_TpiE4_ chromosomes were found as [C/R]_TpiE4_ heterozygotes with only 17% (36/208) hemizygous ([R/Z]_TpiE4_) or homozygous ([R/R]_TpiE4_) for R_TpiE4_. The R_TpiE4_ haplotype was not found in our samples from Karnathaka, India (2018) or China (2019) and was detected only as a heterozygote in the 2019 collections from Myanmar.

**Fig 7:**
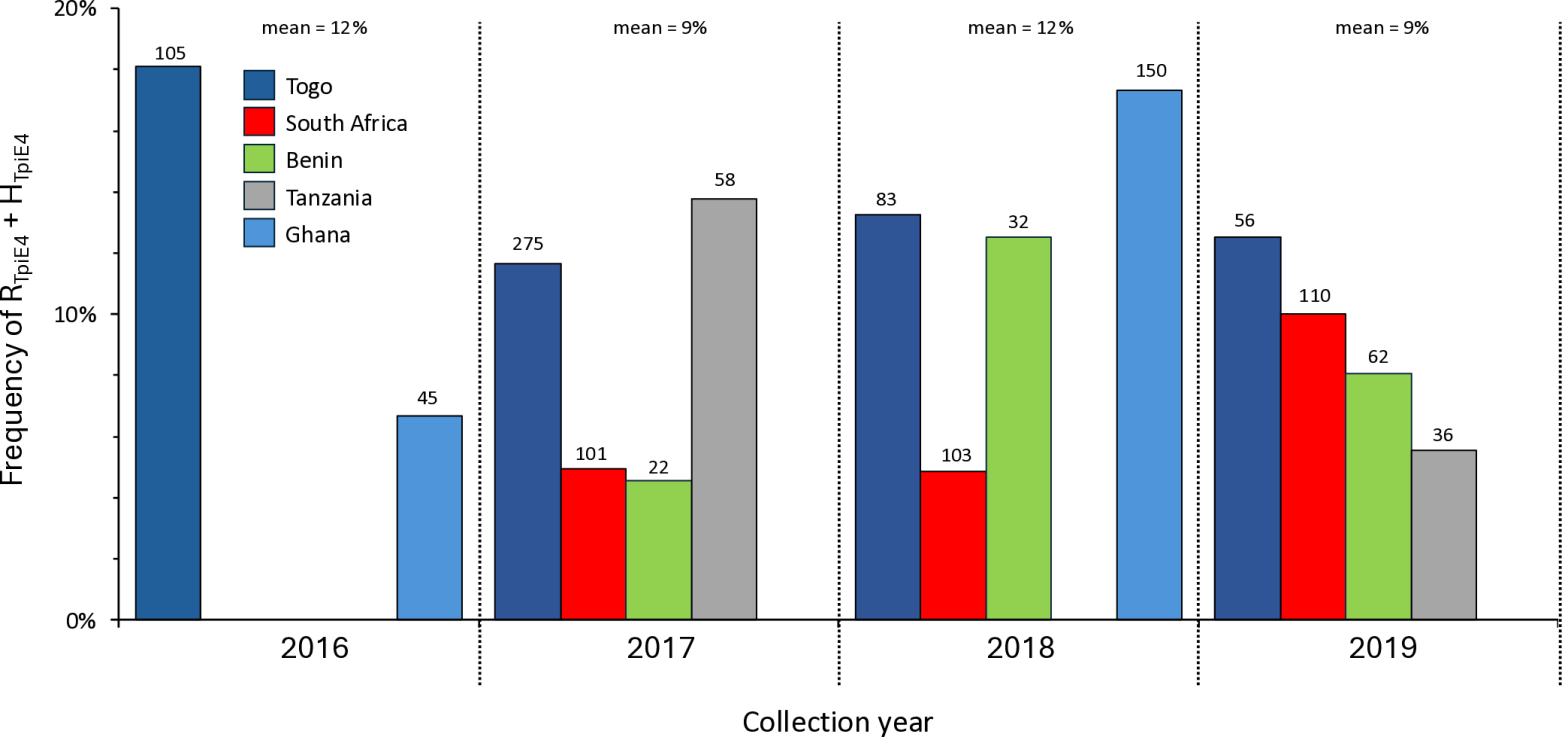
Bar graph describing the percentage of specimens from different countries and years carrying at least one R_TpiE4_ chromosome. Countries are differentiated by color with the number of specimens analyzed above each column. Raw data available in S3 Table.

In five locations, collections were made over multiple years allowing an assessment of how the *Tpi*-based strain profiles changed over time. While variations were observed within years the overall portions remained relatively unchanged, with annual mean R_TpiE4_ + H_TpiE4_ frequencies fluctuating between 9%−12% (Fig 7).

### Equivalence of AfrRa1 and the Zambia-strain and their detection in Mexico

The TpiE4 and TpiI4a200 segments of the Zambia strain (Genbank accession mt767446, [50]) was found to be identical to AfrRa1, consistent with their being part of the same R strain population. In addition, a total of 230 specimens from field collections and colonies representing multiple Mexican states were obtained from a separate study [55]. One specimen from a 2016 Durango field collection expressed the AfrRa1 TpiE4 haplotype, representing the first observation of this sequence in the Western Hemisphere. Additional sequencing demonstrated that it was also identical to AfrRa1 in the TpiI4a200 intron segment. Whether this specimen is from a local endogenous population or is a migrant is unknown.

## Discussion

Understanding the history of FAW introduction into Africa, the composition of the invasive population, and how that population is changing over both time and geography can provide important insights into the current and potential risks posed by FAW to specific crops and how other such invasive events can be mitigated. However, contradictory conclusions from recent population studies using whole genome SNP comparisons have led to confusion in two important areas. In one, the strain composition of the Eastern Hemisphere FAW populations has been characterized as being entirely of the C strain [50,66,67], primarily interstrain hybrids [2], or of no strain (as defined by *Tpi* markers) [40]. One likely explanation for these disagreements is the significant but still restricted cross-hybridization that can occur between the strains [18,23] and the primacy of the Z-chromosome in determining strain identity [24,25,41].

A consequence of partial reproductive isolation is that the two strain populations will undergo broad genomic differentiation but that the magnitude of the genetic differences and the composition of strain-specific polymorphisms will vary by population. This is because most of the strain-biased SNPs will result from random genetic hitchhiking and drift and therefore exhibit high variability. In contrast, SNPs linked to alleles required for strain identity will be directly selected for and thereby show strain-specificity in all populations. Currently, only the Z-chromosome has shown consistent genetic differentiation between strains in multiple studies by different groups and samplings [24,31,36,38,68,69]. In contrast, while strain-specificity has been reported for several autosomes [28,31,38,49,69], in no case has the identification of a specific autosome as strain biased been conclusively confirmed in a separate study. This absence of replicability suggests that the strain-specificity of the autosomal sites are limited to the collection sampled rather than being generally representative of the species.

Fiteni et al (2022) is particularly instructive on this issue. They found that in a sampling of FAW from Florida corn and pasture habitats there was significant genetic differentiation in the autosomes between the strains, which was interpreted as representing differentiation at the genomic level. However, one of the most strain-specific autosomes identified in that study (chromosome 12) has not shown similar strain-specificity in surveys by other groups. This is consistent with genetic differentiation in the autosomes occurring stochastically with few, if any, autosomal sites being required for strain identity as defined by the plant host use phenotype [19]. The expected consequence is genetic differentiation of the autosomes between strains that differ in magnitude and SNP composition between populations.

Given the likelihood of substantial regional variation, autosomal SNPs are of limited usefulness for strain identification. As such, their inclusion in whole genome analysis could obscure the accurate discrimination of the host strains by Z-linked SNPs such as *Tpi*, whose legitimacy as a marker for strain identity has been demonstrated by multiple assays of populations from both North America and South America that show a consistent correspondence with plant host use. At this time, the same criterium has not been met by any whole genome-based methodology for strain identification.

A second area of disagreement concerns the direction of FAW dissemination across the Eastern Hemisphere, with genetic support presented for both an Asia to Africa [2,40] and an Africa to Asia migration [53,70]. This conflict likely results from the high genetic variation [27,71,72] and long-distance mobility [73] characteristics of FAW, with the former requiring large sample sizes to demonstrate reproducible SNP profiles while the latter means that any field collection may be from a transient rather than permanent local population. Either of these factors can compromise the reproducibility of extrapolated FAW population structure and migratory pathways. Therefore, descriptions of population structure that have not been demonstrated to persist over time (ideally over multiple years) cannot be assumed to represent the distribution of permanent or recurring populations and so have limited usefulness in extrapolating migration behaviors.

In recognition of these issues, the objective of this study was to test if our earlier descriptions of the Eastern Hemisphere FAW are reproducible when compared with additional sampling and analysis with a new (TpiI4a200) genetic marker. Of particular interest was to confirm both the R strain identification of AfrRa1, which would make host plants associated with this strain at risk in Africa [12], and the suggestion of an additional introduction of FAW into Africa from a new Western Hemisphere source [15].

The analyses supported the past conclusions and can be summarized by three significant findings. First is evidence for the additional FAW introduction event in the form of three C_TpiE4_ (AfrCa1d, e, f) and one R_TpiE4_ (AfrRa2) haplotype not previously seen in Africa. These were detected at approximately the same time and location (Oct-Dec, 2018 collections from Vogan, Togo) as observations of unusually high frequencies of the CSh1 and CSh2 haplotypes that were previously conjectured to have come from a location outside of Africa where both are prevalent (e.g., South America, Mexico, or Texas) [15]. Evidence of new FAW variants is a concern given observations of regional populations in the Americas that differ in host usage [19,74], mating behavior [29,32,33], migratory behavior [75], and pesticide resistance [76–78]. This means there is potential for significant changes in the crops impacted and the effectiveness of specific pesticides with continued introductions from the Western Hemisphere. This illustrates the need for continued monitoring for such invasive events, with their potential impact informed by the extrapolation of the likely Western Hemisphere origins.

The second is the identification of AfrRa1 and AfrRa2 as being of the R strain based on phylogenetic comparisons using TpiI4a200. The suggestion that AfrRA2 might have originated from South America is a concern because of indications that the R strain in Florida behaves more like a generalist with respect to host plant usage compared to the more host specific FAW populations in South America [19]. The possibility of region-specific differences in host preferences within strains means that additional introductions into Africa has the potential to significantly alter the risk profile of the African R strain population.

The third finding is that AfrRa1 and the Zambia strain share an identical TpiI4a200 sequence, which is strong evidence that the two populations characterized by different laboratories are the same. This indicates a single R_Tpi_ strain population that extended from western Africa to China and southeast Asia during the initial period of FAW detections. The AfrRa1/Zambia strain was detected in Togo and Ghana (western Africa) in 2016, South Africa in 2017, Kenya, the Democratic Republic of Congo, and Zambia (eastern Africa) in 2017, and China in 2019, indicating a west-to-east progression assuming the detection times accurately reflect the movements of the population.

If AfrRa1 and AfrRa2 are of the R strain, then whether one or both become significant pests in Africa will depend on why they are currently so infrequently found in field surveys. AfrRa1 has been present on the continent since 2016 but still represents only about 2% of the sampled homozygous female or hemizygous male population. This could in part be a sampling artifact as the great majority of FAW collections tested were from C strain host plants. Two collections from our data set were from rice habitats, larvae collected from rice in Karnathaka, India and adult males from pheromone traps in rice fields from Kovie, Togo. No significant increase in the frequency of the R_TpiE4_ chromosome was observed in these collections (Fig 4). While suggestive, it should be noted that rice appears to be a secondary host for FAW in the United States as infestations are only sporadically observed. Therefore, the results of our limited sampling on rice are not conclusive. It remains plausible that the R strain is present at higher density in a yet to be identified host plant that is not a significant economic crop and so not routinely scouted.

An alternative explanation is suggested by observations that there are locations in the Western Hemisphere where the C_TpiE4_ strain is present in high numbers but not the R_TpiE4_ strain. In both Arizona (USA) and Ecuador, FAW infestations occur in corn but the R strain has yet to be detected even after multiple years of surveys in R strain preferred habitats in both locations [54,79]. These observations suggest unidentified biological differences between the strains that can significantly impact their geographical distributions. One possibility would be differences in migratory behavior with the R_TpiE4_ strain postulated as being less mobile than the C_TpiE4_ strain. There may also be strain differences in fitness to climatic and other environmental factors or in susceptibility to natural enemies and diseases that is more limiting for the R_TpiE4_ strain. Such factors could explain the variable results obtained from Mexico, where collections from 2013 and 2014 showed no evidence of the R_TpiE4_ strain [56,79], while later collections (post-2017) from different states and locations found a significant fraction [55]. A more compartmentalized or variable R strain distribution would be consistent with reduce mobility and/or greater sensitivity to environmental factors. If such biological differences in the strains exist, it could mean that the R_TpiE4_ strain is having more difficulty adapting to the African environment and, as in parts of the Western Hemisphere, may ultimately have a more limited distribution in the African continent than the C_TpiE4_ strain.

The identification of AfrRa1 as being of the R strain disagrees with Durand et al (2024), which asserted the R-strain was probably not present in the Eastern Hemisphere [66]. This conclusion was based the absence of presumed R strain-associated polymorphisms in the invasive (Eastern Hemisphere) FAW population as described by whole genome SNP comparisons. However, the R strain SNP profile used was generated from only a total of 17 specimens that did not include representatives from the overwintering populations in Texas, Brazil, or Argentina [66] that together probably account for the great majority of FAW in the Western Hemisphere. Given this limited sampling, the collection of SNPs identified as R strain used by Durand et al. (2024) was likely incomplete. Furthermore, the invasive samples analyzed all appear to be collected from C strain host plants and are therefore most likely to be of the C strain. It is therefore unclear why there was any expectation of R strain genetic introgression since none of the samples were behaving as the R strain.

The overall results lead to the following working hypothesis for how FAW became established in Africa. It is postulated that an initial invasive propagule originating from the Florida-Caribbean region was composed of multiple individuals of both strains that included AfrCa1a-c, AfrCa2a-c, and AfrRa1. Cross-hybridization between strains occurred during the invasion process that compromised the strain-specificity of the *COI* marker, with the R_COIB_ C_TpiE4_ configuration becoming dominant (presumably by chance) in the population that moved into the rest of sub-Saharan Africa, India and Myanmar [46,52]. At present, the FAW composition of Africa is primarily C_TpiE4_ strain, with 2% R_TpiE4_ strain, and 10% C_TpiE4_/R_TpiE4_ hybrid.

**Fig 8:**
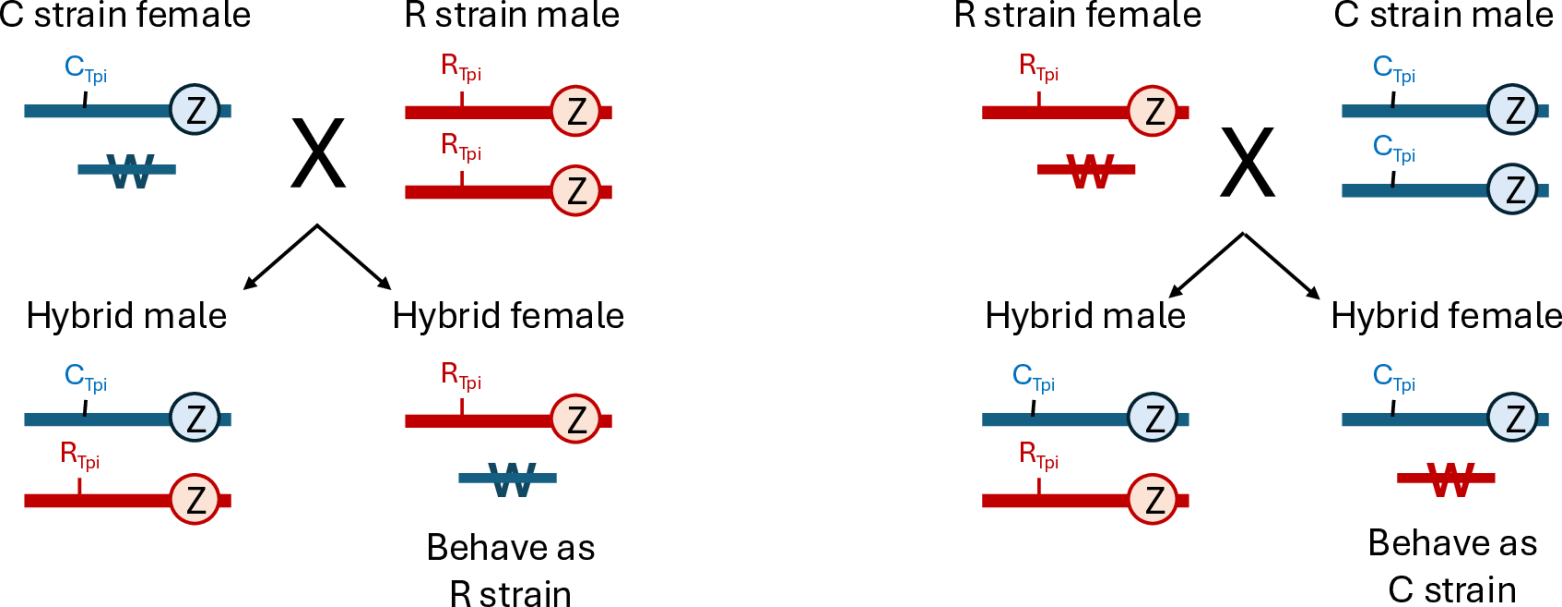
Diagram of reciprocal crosses between strains and their resultant hybrid progeny showing their sex chromosome genotypes. Autosomes are assumed to have no influence on strain identity and are not shown. Chromosomes marked with Z are the Z-chromosome, which carries the genes directing host specificity and the *Tpi* markers. Chromosome with a W is the W-chromosome.

If strain-specific host choice is defined by the Z-chromosome, then only hybrid males (C_TpiE4_/R_TpiE4_) will have an ambiguous strain genotype. However, this occurs in the gender where the only reported strain-specific phenotype is allochronic mating that is only observed in North American populations [21,22,29–31]. The diagnostic host plant phenotype, which depends on where females lay their eggs, should still show the expected strain biases since the hybrid females (Z/W) carry a single Z-chromosome together with a W chromosome assumed to have no genetic influence (Fig 8). Strain identity could be compromised if recombination occurs between the hybrid male Z-chromosomes that mixes the putative strain-determining loci. However, there is preliminary evidence that recombination between the Z-chromosomes in these hybrid males rarely if ever occur [68]. In this scenario, even if the current African FAW population is derived from an ancestral interstrain hybridization, the descendant females should still show the expected strain-specific host plant usage.

In summary, the Africa FAW populations should be considered in a state of flux that while currently a threat primarily to C strain associated host plants could exhibit a significantly wider host range with additional introductions of R strain variants from the Western Hemisphere. There is no reason at present why FAW infestation patterns in Africa and Asia shouldn’t eventually resemble that observed in the American continents without more effective efforts to prevent new introductions. In North America, reports of significant economic damage to R strain associated crops by FAW are generally limited to instances of sporadic “outbreaks” when dense larval populations are suddenly observed. These are typically reported in real time by university extension and government personnel, with examples in forage grasses [80], alfalfa [81], pastures [82], and rice [83]. The biological and environmental conditions that generate such outbreaks are unknown. It is possible that the economic impact of the R strain in Africa and potentially Asia will follow a similar pattern, with periods of low activity interspersed with high-damage outbreak events.

## Supporting information

S1 Fig DNA sequences of TpiE4 and TpiI4a200 segments for the haplotypes found in Africa. (TIF)

S1 Table Data for Fig 3. (DOCX)

S2 Table Data for Fig 6. (DOCX)

S3 Table Data for Fig 7. (DOCX)
