# Supplementary figures and images for: The status in Africa of fall armyworm expressing genetic markers related to infestations of pasture, millet, alfalfa, and rice in the Americas
Source: PLoS One. 2025 Jul 31;20(7):e0329096. doi: 10.1371/journal.pone.0329096 (PMC12312897; doi:10.1371/journal.pone.0329096)

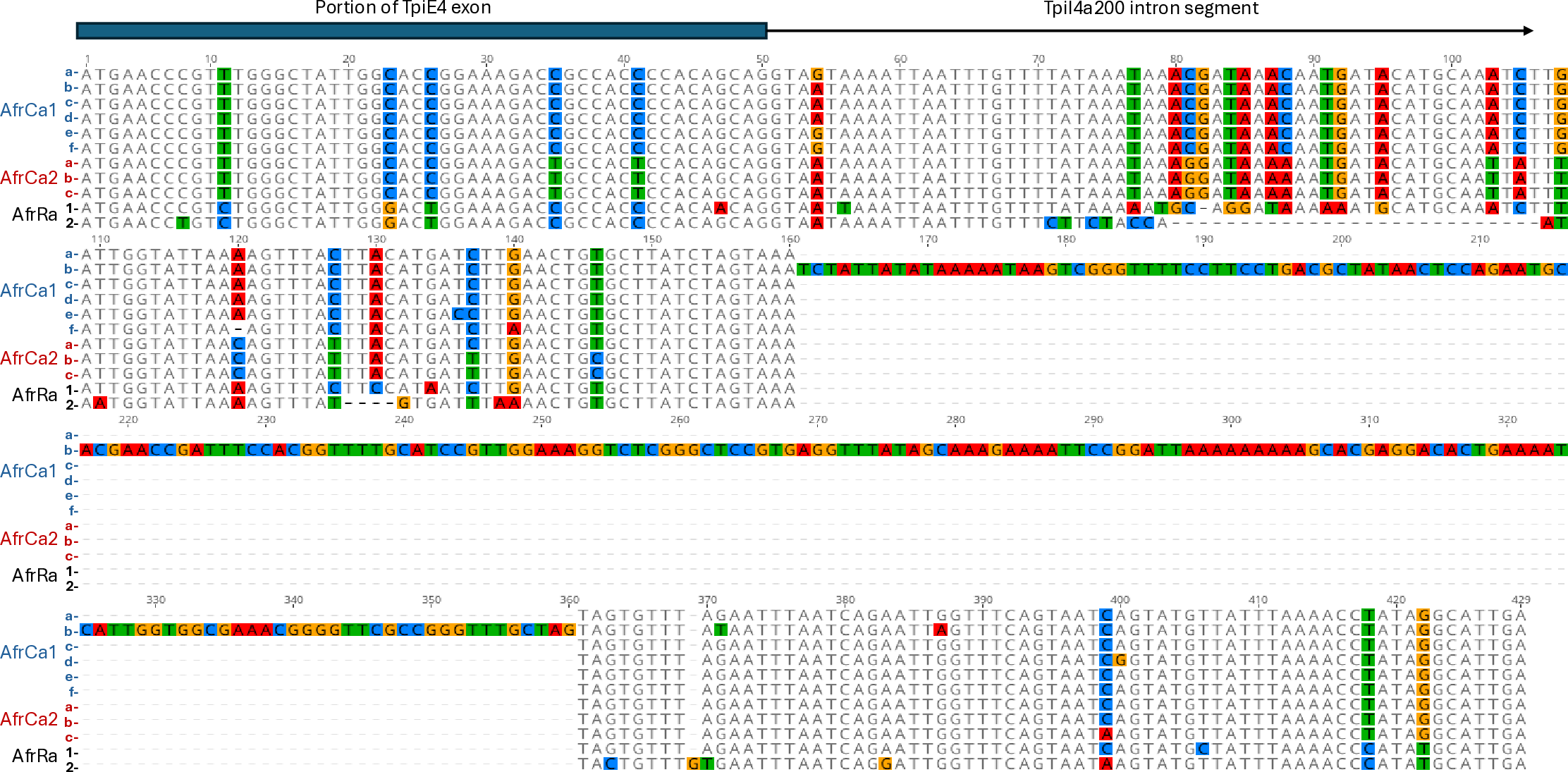

Supplement: S1 Fig — (TIF) [file pone.0329096.s001.tif]
