# Supplementary material for: The status in Africa of fall armyworm expressing genetic markers related to infestations of pasture, millet, alfalfa, and rice in the Americas
Source: PLoS One. 2025 Jul 31;20(7):e0329096. doi: 10.1371/journal.pone.0329096 (PMC12312897; doi:10.1371/journal.pone.0329096)
Supplement: S1 Table — (DOCX) [file pone.0329096.s002.docx]

Supplemental Table S1. Data for Figure 3.

|  | AfrCa1a | AfrCa1b | AfrCa1c | AfrCa2a | AfrCa2b | AfrCa2c | AfrRa1 | otherC | OtherR |
| --- | --- | --- | --- | --- | --- | --- | --- | --- | --- |
| 2016 | 8 | 0 | 71 | 0 | 5 | 17 | 5 | 0 | 0 |
| 2017 | 57 | 1 | 290 | 3 | 30 | 89 | 8 | 0 | 0 |
| 2018 | 31 | 0 | 140 | 1 | 9 | 32 | 4 | 3 | 1 |
| 2019 | 17 | 0 | 106 | 1 | 5 | 33 | 14 | 0 | 0 |
