# Supplementary material for: The status in Africa of fall armyworm expressing genetic markers related to infestations of pasture, millet, alfalfa, and rice in the Americas
Source: PLoS One. 2025 Jul 31;20(7):e0329096. doi: 10.1371/journal.pone.0329096 (PMC12312897; doi:10.1371/journal.pone.0329096)
Supplement: S2 Table — (DOCX) [file pone.0329096.s003.docx]

Supplemental Table S2. Data for Figure 6.

|  | TpiC | TpiR | TpiH | Total |
| --- | --- | --- | --- | --- |
| Ben | 106 | 4 | 6 | 116 |
| Bur | 44 | 0 | 7 | 51 |
| Cam | 148 | 3 | 18 | 169 |
| CAR | 36 | 0 | 1 | 37 |
| Cha | 16 | 0 | 4 | 20 |
| Ind corn | 18 | 0 | 0 | 18 |
| Ind rice | 9 | 0 | 0 | 9 |
| CV | 24 | 0 | 1 | 25 |
| DRCn | 25 | 1 | 3 | 29 |
| DRCs | 67 | 1 | 4 | 72 |
| Gha | 166 | 4 | 25 | 195 |
| Ken | 31 | 1 | 9 | 41 |
| Mya | 18 | 0 | 3 | 21 |
| Nga | 13 | 0 | 3 | 16 |
| SAf | 285 | 9 | 15 | 309 |
| Sat | 21 | 0 | 6 | 27 |
| Sen | 22 | 2 | 5 | 29 |
| Tog corn | 319 | 9 | 24 | 352 |
| Tog rice | 59 | 5 | 3 | 67 |
| Zam | 75 | 0 | 9 | 84 |
| Tan | 84 | 0 | 10 | 94 |
