# Supplementary material for: The status in Africa of fall armyworm expressing genetic markers related to infestations of pasture, millet, alfalfa, and rice in the Americas
Source: PLoS One. 2025 Jul 31;20(7):e0329096. doi: 10.1371/journal.pone.0329096 (PMC12312897; doi:10.1371/journal.pone.0329096)
Supplement: S3 Table — (DOCX) [file pone.0329096.s004.docx]

Supplemental Table S3. Data for Figure 7.

| Collection | Year | C_TpiE4_ | R_TpiE4_ | H_TpiE4_ | Total | (R_TpiE4_+ H_TpiE4_)/Total |
| --- | --- | --- | --- | --- | --- | --- |
| Tog16 | 2016 | 86 | 3 | 16 | 105 | 0.18 |
| Gha16 | 2016 | 42 | 2 | 1 | 45 | 0.07 |
| Tog17 | 2017 | 243 | 3 | 29 | 275 | 0.12 |
| SAf17 | 2017 | 96 | 2 | 3 | 101 | 0.05 |
| Ben17 | 2017 | 21 | 0 | 1 | 22 | 0.05 |
| Tan17 | 2017 | 50 | 0 | 8 | 58 | 0.14 |
| Tog18 | 2018 | 72 | 2 | 9 | 83 | 0.13 |
| SAf18 | 2018 | 98 | 0 | 5 | 103 | 0.05 |
| Ben18 | 2018 | 28 | 1 | 3 | 32 | 0.13 |
| Gha18 | 2018 | 124 | 2 | 24 | 150 | 0.17 |
| Togo 2019 | 2019 | 49 | 5 | 2 | 56 | 0.13 |
| SAf2019 | 2019 | 99 | 5 | 6 | 110 | 0.10 |
| Ben19 | 2019 | 57 | 3 | 2 | 62 | 0.08 |
| Tanz2019 | 2019 | 34 | 0 | 2 | 36 | 0.06 |
